# Investigation and Characterization of Synthesis Conditions on Sucrose-ammonium Dihydrogen Phosphate (SADP) Adhesive: Bond Performance and Chemical Transformation

**DOI:** 10.3390/ma12244078

**Published:** 2019-12-06

**Authors:** Shijing Sun, Min Zhang, Kenji Umemura, Zhongyuan Zhao

**Affiliations:** 1 College of Material Science and Engineering, Nanjing Forestry University, Nanjing 210037, China; sunsj-611@163.com; 2 Laboratory of Sustainable Materials, Research Institute for Sustainable Humanosphere, Kyoto University, Gokasho, Uji, Kyoto 611-0011, Japan; zhang888@rish.kyoto-u.ac.jp (M.Z.); Umemura@rish.kyoto-u.ac.jp (K.U.); 3 College of Furnishings and Industrial Design, Nanjing Forestry University, Nanjing 210037, China

**Keywords:** plywood, sucrose, ammonium dihydrogen phosphate, eco-friendly adhesive

## Abstract

Sucrose is one of the most abundantly available renewable chemicals in the world, and it is expected to be utilized as a raw material for wood-based material products. Herein, a novel adhesion system that was based on sucrose and ammonium dihydrogen phosphate (ADP) was synthesized into an adhesive with 80% solid content, and this eco-friendly was utilized on the fabrication of plywood. The effects of the synthesis conditions on the plywood bond performance and synthesis mechanism were investigated. The optimal synthesis conditions were as follows: the mass proportion between sucrose and ADP was 90/10, the synthesis temperature was 90 °C, and the synthesis time was 3 h. The bonding performance of the plywood that was bonded by optimal SADP adhesive satisfied the GB/T 9846-2015 standard. The chemical analysis was performance tested by using High-Performance Liquid Chromatography (HPLC), Attenuated Total Reflection-Fourier Transform Infrared Spectra (ATR-FTIR), and Pyrolysis Gas Chromatography and Mass Spectrometry (Py-GC/MS) to understand the chemical transformation during the synthesis process. The chemical analysis results confirmed that the hydrolysis and conversation reaction of sucrose occurred in the synthesized SADP adhesive, and ADP promoted the pyrolysis efficiency of sucrose.

## 1. Introduction

With the decrease of fossil resources, the development of bio-based materials has attracted more attention in the material science and technology [1,2,3,4,5]. Actually, some wood-based materials are widely utilized in our living environment (such as particleboard, plywood, and fiberboard), and these materials are commonly fabricated by synthesis adhesives (such as urea-formaldehyde [6], melamine-formaldehyde [7], phenol-formaldehyde [8,9], etc.). Attributed to the low-cost and excellent properties of these synthetic adhesives, it is hardly to develop the bio-based adhesives that act as consummate substitutes in the wood panel industry [10]. However, the worry about formaldehyde emission from the panels is becoming a most concerning issue for the consumers and regulatory, as an inevitable trend, the legislations related both work environment and final product emissions have steadily become stricter over time. Therefore, researchers are devoting their efforts to utilize nontoxic feedstock as raw materials for the production of adhesives. Lignin, soybean protein, starch, and more are examples of these raw materials [11,12,13,14,15].

Sucrose is one of the most abundantly available renewable resources in the world. The potential of this almost ubiquitous, the natural product as a chemical raw material has been extensively explored and widely used in the beverage and food industries [16,17,18]. Hence, we developed a series of sucrose-based wood adhesives, such as sucrose-citric acid [19,20], sucrose-tannin [21,22], and sucrose-acid-tannin adhesives [23], when considering the renewability and reactivity of sucrose. In these researches, sucrose and other soluble compounds were dissolved in the distilled water at room temperature and applied to manufacture the particleboard. The reaction mechanism of these adhesives confirmed that the sucrose convert to 5-hydroxymethylfurfural (5-HMF) and react with other components to form carbonyl groups and ether linkages, in which 5-HMF is considered to be one of the most important sucrose-derived products to gain the good bond performance of particleboard. However, these reports found that the prepared adhesives required very low pH values, which was due to acidic catalysis of sucrose to form 5-HMF without significant addition of temperature, while these low pH requirements might pose corrosion risks of the metal used in the manufacturing process of wood-based materials. Therefore, we shifted focus to use phosphate as catalyst to promote the conversion of sucrose and develop a novel adhesive composed by sucrose and ammonium dihydrogen phosphate (ADP) for particleboard [24].

The previous researches above have utilized the solubility and reactively of sucrose to prepare a low viscosity eco-friendly adhesive for particleboard. Based on the sucrose chemistry, there is another noteworthy property of sucrose that a high concentration amorphous solution with relatively low viscosity could be obtained by heating sucrose with little distilled water [16,17]. This provided the possibility of synthesizing a sucrose-based adhesive with higher solid content and viscosities that are tolerable for wood product manufacturing. In this study, we synthesized an intriguing new sucrose-based adhesive for the plywood when considering the good bond performance and low pH value of sucrose-ammonium dihydrogen phosphate adhesion system, and the effects of synthesis conditions on the bond performance and synthesis mechanism were investigated.

## 2. Materials and Methods

### 2.1. Materials

Sucrose (analytical-grade reagent) and ammonium dihydrogen phosphate (analytical-grade reagent) were purchased from Sinopharm Chemical Reagent Co., Ltd., (Shanghai, China). Each reagent was vacuum-dried at 60 °C until constant mass prior to usage in experiments. The poplar veneers were obtained from Zuogezhuang, China.

### 2.2. Synthesis of Adhesives

**Figure 1:**
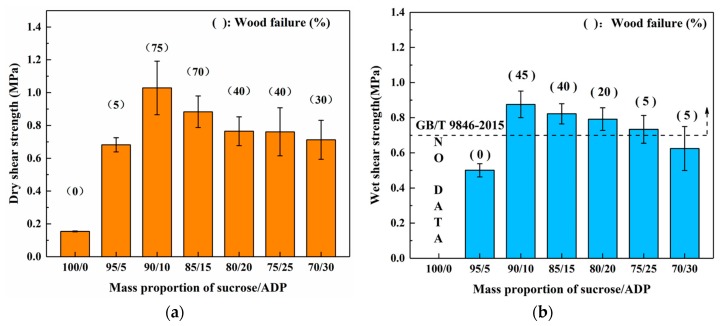
Effects of mass proportion between sucrose and ammonium dihydrogen phosphate (ADP) on the bond performance of plywood (**a**) dry shear strength, and (**b**) wet shear strength.

Sucrose and ammonium dihydrogen phosphate (ADP) were mixed at different proportions, and they were poured into a three mouthed bottle with distilled water to synthesize Sucrose-Ammonium Dihydrogen Phosphate (SADP) adhesives with 80 wt% solid content. Three groups of adhesives were synthesized to investigate the effects of proportion, synthesis temperature, and synthesis time on the bond performance of plywood, and Table 1 shows detailed information of the synthesis condition of each group. All of the synthesis processes were conducted in an oil bath and under 180 rpm/min stirring. The pH of the adhesives were measured at 30 °C using a Leici pH meter PHBJ-206 (Leici, Shanghai, China). The viscosities of the adhesives were measured while using a HAAKE rotational rheometer MA S60 (HAAKE CO., Karlsruhe, Germany). Specifically, 2 mL of each adhesive were loaded onto a flat plate set at 30 °C. The measuring geometry (C60 2°/Ti-02170027) was lowered next, and the testing mode was CR while using 100/s shear rate. Each viscosity test experiment lasted 300 s until the viscosity values stabilized. A total of 80 viscosity measurements were acquired over the duration of analysis, while the final viscosity was defined as the average values of the last 40 data points. Table 1 shows the results of pH and viscosity. Figure 1 shows the appearance of synthesized SADP adhesives. All of the synthesized SADP adhesives were sealed and stored at room temperature for at least three days before further research was conducted involving them.

### 2.3. Bond Performance

#### 2.3.1. Manufacture of Plywood

The synthesized SADP adhesives were used to manufacture a three-layer plywood (300 mm × 300 mm), the bond performance of which was evaluated. The moisture content and thickness of the veneers were 9.8–11% and 1.5 mm, respectively. SADP adhesives were applied to the core veneer at a spread rate of 140 g/m^2^ for a single veneer surface. The coated veneer was stacked between two uncoated veneers, so that the grain direction of both adjacent veneers was perpendicular to each other. All of the assembled three-layered plywood samples with each SADP adhesive were hot-pressed at 170 °C for 7 min.

#### 2.3.2. Shear Strength Measurement

The prepared plywood was cut into standard tensile shear test specimens, according to China National Standards (GB/T 9846.7-2004). Twelve plywood specimens (10 cm × 2.5 cm) were cut from each manufactured plywood and six specimens were submerged into water at 63 ± 2 °C for 3 h, and the tensile shear strength of the plywood was then measured at dry and wet condition under a loading rate of 1.0 mm/min. Each plywood test was carried out in twelve replications, and the average values, standard deviations, and average wood failure were calculated. Statistical significance was considered for *p* values < 0.05.

### 2.4. Chemical Analysis of Synsized SADP Adhesives

#### 2.4.1. High-Performance Liquid Chromatography (HPLC) Analysis

The main chemical composition of the liquid SADP adhesives from Groups 2 and 3 were measured while using Agillent 1260 high-performance liquid chromatography (HPLC) (Agillent Technologies Inc., Santa Clara, CA, USA, USA). The accurate results, before the measurement, and the adhesive solutions were diluted 300 times, and the final contents were calculated by multiplying by 300, due to the concentration of SADP adhesives being too high to measure. The HPLC system was equipped with an HPX-87H lon exclusion column (300 mm × 7.8 mm) (Bio-Rad, Hercules, CA, USA), degasser, pump, and refractive index (RI) detector. HPLC grade milli-Q water was used as the eluent at a flow rate of 0.6 mL/min at a column temperature of 55 °C.

#### 2.4.2. Attenuated Total Reflection-Fourier Transform Infrared Spectra (ATR-FTIR) Analysis

The ATR-FTIR spectra were acquired to probe for chemical changes between SADP 100/0 and SADP 90/10 (sucrose/ADP mass proportion), which synthesized at optimal synthesis conditions. Infrared spectra of freeze dried adhesives were obtained while using an ATR-FTIR spectrophotometer (Nicolet iS10, Thermo Fisher Scientific, Waltham, MA, USA), and recorded with an average of 32 scans at a resolution of 4 cm^−1^.

#### 2.4.3. Pyrolysis Gas Chromatography and Mass Spectrometry (Py-GC/MS)

Py-GC/MS was employed to clarify the effects of ADP addition on the synthesized adhesives. The synthesized SADP adhesives with 100/0 and 90/10 (sucrose/ADP mass proportion) were freeze dried to obtained uncured adhesives, and the volatile organic compounds (VOCs) of SADP adhesives during the curing process were measured by a Py-GC/MS system (GCMS-QP2010, Shimadzu Co., Ltd., Kyoto, Japan). Approximately 1 mg samples was placed in a small cup and was pyrolyzed at 170 °C with 60 s by Multi-Shot pyrolyzer (EGA/PY-3030d, Frontier Laboratories Ltd., Koriyama, Japan), and the pyroprobe interface temperature was set at 220 °C. The column was an Ultra ALLOY-5 capillary column (30 m × 0.25 mm i.d., 0.25 µm film thickness, Frontier Laboratories Ltd., Fukushima, Japan). The initial temperature of the column was set at 37 °C for 2 min, and the temperature was then increased to 100 °C with a rate of 30 °C/min immediately, and the temperature was further increased to 220 °C at a rate of 15 °C/min and kept at 220 °C for 1 min. Helium (99.999%) was used as a carrier gas with a column flow of 1.0 mL/min, and all the injections were in splitless mode. Capillary direct MS interface temperature was 320 °C, the ion source temperature was 200 °C, and the carrier inlet pressure at 50 kPa. The mass spectrometer was operated in EI mode at 70 eV, and the analyzed range was 50–600 m/z with a scan speed of 1250 amu/s. The pyrolysis products were identified while using the NIST 08 mass spectral library, and the volatile components with a greater than 90 similarity index (SI) or the compounds with the highest SI value (the SI value of all the identified compounds in one peak lower than 90 condition) were selected.

## 3. Results and Discussion

### 3.1. Effects of Synthesis Conditions on Viscosity, pH Values, and Crystallization of SADP Adhesives

Table 1 shows the basic characteristics of SADP adhesives that synthesized at different conditions, and all of the results were measured after storing for three days at room temperature. In all groups, viscosity and pH values both decreased by increasing the content of ADP. In regard to the viscosity, the heating treatment caused the hydrolysis of sucrose into monosaccharide and formed amorphous substances that contained monosaccharide and sucrose with high viscosity [25,26,27]. However, as to maintaining this heating status, the monosaccharide would convert to some micromolecule compounds (such as furfural and 5-HMF [28]) under the catalysis of salt or acid [29,30], and this conversion reaction is accelerated by increasing the heating temperature and time. Therefore, the viscosity exhibited a reducing trend in each Group, definitely, the trend in Group 1 was also due to the dilution effect of ADP. The change of pH values induced possible effects by the hydrolysis of ammonium ion [31], which resulted the concentration increases of hydrogen ion. The crystallization could be observed in sucrose (100/0), and this was due to the solution with 80% solid content that achieved the supersaturated state when the temperature decreased from 90 °C to room temperature. However, the crystalline components were not observed from other adhesives mixed with sucrose and ADP, which indicated that some conversation reaction occurred and formed amorphous solutions, which prevented the crystallization [32].

### 3.2. Bond Performance

#### 3.2.1. Effects of Mass Proportion between Sucrose and ADP

Three-ply plywood were fabricated while using the synthesized SADP adhesives with various mass proportion to investigate the effects of mass proportion between sucrose and ADP on the bond performance, and Figure 1a,b show the results of dry and wet shear strength, respectively. In the dry shear strength, when the mass proportion of ADP increased from 0 to 10%, the boards exhibited a positive correlation between mechanical properties and mass ratio of ADP. However, a slight decrease of dry shear strength and wood failure was observed while increasing the mass proportion of ADP equal to and higher than 15%. It is possible that this phenomenon was caused by an effect of redundant ADP on the curing rate of SADP adhesive. Regarding wet shear strength (Figure 1b), a trend that was similar to what was observed for dry shear strength could be found. It is worth noting that the board formulated with pure sucrose exhibited dry shear strength of 0.15 MPa, and the board destroyed after the immersion treatment. This indicates that essentially low water resistance is gained solely upon sucrose, and the addition of ADP contributed to the increasing of mechanical properties and water resistance. The wet shear strength satisfied to the requirement of China National Standard (GB/T 9846-2015) when the plywood bonded by SADP adhesives with ADP content in the range of 10–25%. The highest shear strength and wood failure values were obtained from the board bonded with SADP 90/10 (0.88 MPa, 45%); therefore, the mass proportion between sucrose and ADP at 90/10 was considered as one of the optimal synthesis conditions that will be used for further research.

#### 3.2.2. Effects of Synthesis Temperature

**Figure 2:**
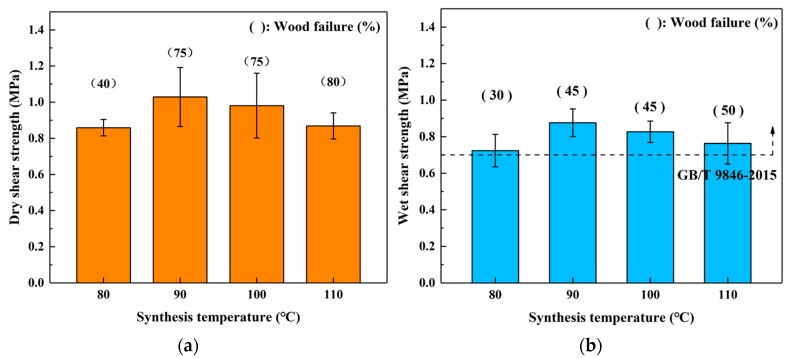
Effects of synthesis temperature of Sucrose-Ammonium Dihydrogen Phosphate (SADP) adhesives (synthesis time: 3 h) on the bond performance of plywood (**a**) dry shear strength, and (**b**) wet shear strength.

Figure 2 shows the bond performance of SADP adhesives that were synthesized with 90/10 mass proportion at different synthesis temperatures. The profiles that were visualized in both dry shear strength (Figure 2a) and wet shear strength (Figure 2b) exhibited a similar trend, in which the bond strength increased in the synthesis temperature region of 80–90 °C, and then deteriorated from 100 to 110 °C. However, the wood failure of both dry and wet conditions showed a positive relationship with synthesis temperature. When considering the pH values of synthesized SADP adhesives in Group 2 (Table 2), this performance result indicated that the decreasing of bond strength was possibly attributed to the deteriorating of wood veneer strength at high pressing temperature and acid environment. The wet shear strength of all the samples that were bonded by SADP adhesives satisfied the requirement of China National Standard (GB/T 9846-2015), and the maximum value was 0.88 MPa, as obtained from the SADP adhesive that was synthesized at 90 °C.

#### 3.2.3. Effects of Synthesis Time

**Figure 3:**
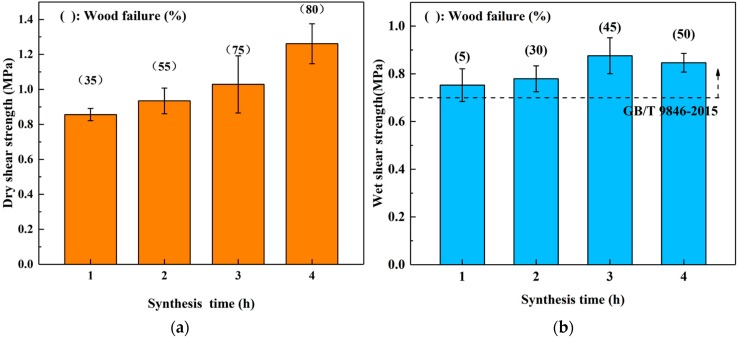
Effects of synthesis time of SADP adhesives (synthesis temperature: 90 °C) on the bond performance of plywood (**a**) dry shear strength, and (**b**) wet shear strength.

The plywoods were manufactured by the adhesives that were synthesized with 90/10 mass proportion at 90 °C for 1–4 h for investigating the effects of synthesis time on the bond properties of SADP adhesives, and Figure 3a,b, show the dry and wet shear strength, respectively. In the dry shear strength, both bond strength and wood failure exhibited a positive correlation with synthesis time, implying that the synthesis time caused a pronounced effect on the bond properties of SADP adhesive. Regarding the wet shear strength, the average values exhibited a rising trend as the synthesis time was increased from 1–3 h, and a slightly decreased was found from the synthesis time at 4 h. However, variance analysis (ANOVA) revealed no significant (*p* > 0.05) difference of wet shear strength between the plywoods that were manufactured with SADP adhesives synthesis for 3 and 4 h. The wet shear strength of all the specimens fulfilled the amount allowed by China National Standard (GB/T 9846-2015) of second class plywood (>0.7 MPa), and the maximum wet shear strength of the plywood was derived from the board that was bonded with SADP adhesive synthesized for 3 h (0.88 MPa).

The optimal mass proportion, synthesis temperature, and time for the tested SADP adhesives was concluded to be sucrose/ADP at 90/10, 90 °C and 3 h, respectively, based on the holistic performance of the plywood bonded with SADP adhesives that were synthesized with different conditions. However, the hot pressing experiments in this research were carried out with relatively higher parameters (170 °C and 7 min) to investigate the bond performance of SADP adhesive at sufficient curing condition, regardless of the SADP adhesive that was synthesized under the optimal conditions.

### 3.3. Chemical Analysis

#### 3.3.1. HPLC

HPLC were used for the measurement to investigate the bond performance of SADP adhesive at sufficient curing condition, and Table 2 shows the results. When considering the mass proportion of sucrose and ADP changed in Group 1, which caused a pronounced effect on the content of 5-HMF; therefore, we just show the results from Group 2 and 3. These two Groups both exhibit a rising trend of 5-HMF content by adding the synthesis temperature and time, respectively. Based on the results of pH values in the synthesized adhesives shown in Table 1, this observation could be explained by the occurrence of sucrose hydrolysis during the heating process, and the generated monosaccharides (glucose and fructose) then further dehydrated and formed 5-HMF. When compared with the bond performance, the values of wood failure (regardless at dry and wet conditions) exhibited a positive correlation with 5-HMF content increasing, which indicated that the content of 5-HMF influenced the curing process of SADP adhesives.

#### 3.3.2. ATR FT-IR

**Figure 4:**
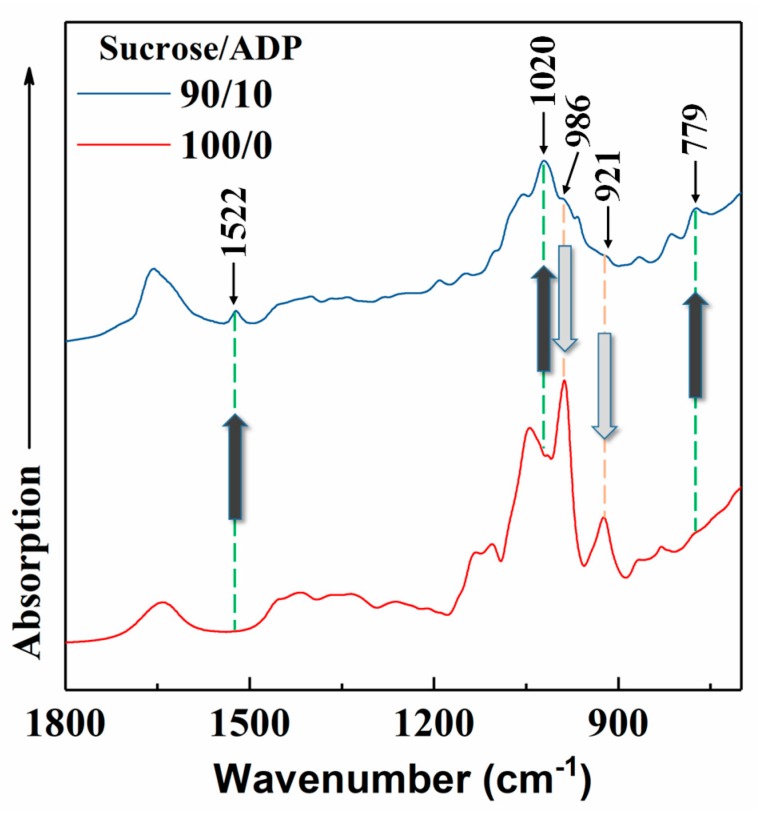
Fourier Transform Infrared Spectra (FT-IR) spectra of sucrose only (100/0) and SADP adhesive (90/10) which synthesized at optimal conditions.

Figure 4 shows the absorption bands that were exhibited by the freeze dried sucrose only (100/0) and SADP (90/10) adhesives that had been synthesized at optimal synthesis conditions (synthesis temperature was 90 °C and synthesis time was 3 h). When comparing to the sucrose only condition, it was observed that two peaks located at 986 and 921 cm^−1^ decreased in signal intensity as an addition of ADP, and these peaks were derived from the products of sucrose hydrolyzation that were ascribed to –OH groups and pyranose ring, respectively. The decreasing of these two functional groups indicated that the addition of ADP promoted the dehydration reaction of sucrose hydroxylation products during the synthesis process. Meanwhile, the absorption bands at 1522, 1020, and 779 cm^−1^ increased by adding ADP in the synthesis system. The peaks at around 1522 and 779 cm^−1^ were attributed to C=C stretching vibration and the CH=CH of the furan ring [33,34], and this observation serves as further proof of the production of 5-HMF. Another new peak that was located at around 1020 cm^−1^ was considered as the ether linkage C–O–R [35], which was possibly due to the formation of oligosaccharide during synthesis treatment.

Judging from the results of HPLC and ATR FT-IR analysis and while combining with the results of pH and viscosity, the synthesis process could be described, as follows: sucrose hydrolysis to glucose and fructose that was catalyzed by ammonium and then a conversation reaction that occurred on the monosaccharide that formed 5-HMF. When considering that the pH of the solution was decreased during the synthesis process, it seems that some acid was generated by heating the solution, which was possible due to the consumption of ammonium (the pyrolysis of ammonium ion [31]). In addition, C–O–R seems to form and link some oligosaccharides. However, the viscosity of the solution decreased with increasing the synthesis time and temperature, which indicated that the molecule weight of the synthesized ADP adhesive decreased during the synthesis process. Therefore, the synthesized SADP adhesive was considered as a mixture of monosaccharide, 5-HMF, acid, oligosaccharide, and others.

#### 3.3.3. Py-GC/MS

**Figure 5:**
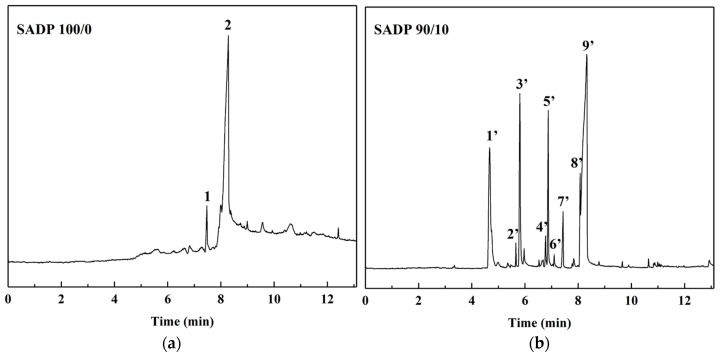
Gas Chromatography/Mass Spectrometry (GC/MS) chromatogram of the evolved gas derived from SADP adhesives (**a**) SADP 100/0 (**b**) SADP 90/10 heated at 170 °C for 60 s.

We chose to produce Py-GC/MS chromatogram of the synthesized SADP 100/0 and SADP 90/10 adhesives to investigate influence of ADP on the molecule transformation of sucrose during the curing process. Figure 5 shows the GC/MS chromatogram of the evolved gas by heating the uncured adhesives at 170 °C. Table 3 reports the identified volatile components with a greater than 90 similarity index (SI) or the compounds with highest SI value (the SI value of all the identified compounds in one peak lower than 90 condition). The pyrogram of SADP 100/0 exhibits two main pyrolysis products: 4H-Pyran-4-one,2,3-dihydro-3,5-dihydroxy-6-methyl- (DDMP) and 5-Hydrxoymethylfurfura (5-HMF), both of which are the characteristic products by heating saccharide [36,37,38]. The profiles visualized in Figure 5b exhibited a significant variation that was ascribed to the addition of ADP when compared with the chromatogram of SADP 100/0, and even more chemical compounds were observed from the evolved gas by heating SADP 90/10. It could be found that most of these substances were furan, pyran, and ketone related compounds, which was typically produced by the dehydration reaction or the hydrolysis of sucrose [39,40,41,42], and this demonstrated that ADP remarkably reduced the sucrose pyrolysis temperature. An exception was observed in the peak number of 5′ (Furan-2-carbohydrazide) was possibly produced during the heating treatment, which seems to demonstrate that the inorganic ADP converted to the organic substances during the curing reaction.

## 4. Conclusions

In this study, a novel eco-friendly wood adhesive that contains sucrose and ammonium dihydrogen phosphate (ADP) was synthesized for fabricating plywood. The effects of synthesis conditions on the bond performance and the chemical analysis of synthesized adhesives were investigated. When the SADP adhesive that was synthesized with 90/10 (sucrose/ADP) mass proportion with 90 °C for 3 h, the bond properties of the final plywood fulfilled the requirement of the GB/T 9846-2015 standard. The results from HPLC elucidate that the addition of ADP promoted the conversion of sucrose to 5-HMF; ATR FT-IR spectra further proved the production of 5-HMF, and some of the formed polymers seemed to contain C–O–R linkage. Py-GC/MS studied the influence of ADP on the molecule transformation of sucrose during the curing process; the addition of ADP accelerated the thermal pyrolysis of sucrose.

The results of this research provided a possibility to synthesize an eco-friendly sucrose-based adhesive for plywood; further researches for investigating the optimal hot pressing conditions, curing behavior, and mechanism are expected.

## Figures and Tables

**Table 1:** Synthesis conditions, results of viscosity, pH values, and precipitation effect.

Groups	Mass Proportion (Sucrose/ADP)	Synthesis Temperature (°C)	Synthesis Time (h)	Solid Content (%)	Viscosity (mPa·s)	pH	Whether Contain the Precipitation after 3 Days Storing
Group 1	100/0	90	3	80	1770	5.1	Yes
	90/10				826.7	3.7	NO
	85/15				784.9	3.3	NO
	80/20				557.6	3.1	NO
	75/25				333.8	2.9	NO
	70/30				252.5	2.5	NO
Group 2	90/10	80	3	80	911.5	4.0	NO
		90			826.7	3.7	NO
		100			472.9	3.1	NO
		110			393.2	2.5	NO
Group 3	90/10	90	1	80	1020.4	4.6	NO
			2		877.0	4.1	NO
			3		826.7	3.7	NO
			4		427.3	2.5	NO

**Table 2:** Chemical composition of synthesized SADP adhesives in Groups 2 and 3.

Groups	Sucrose-ADP	Synthesis Temperature (°C)	Synthesis Time (h)	5-HMF (g/L)
Group 2	90/10	80	3	7.3
		90		31.1
		100		35.6
		110		42.8
Group 3	90/10	90	1	6.7
			2	22.3
			3	31.1
			4	44.5

**Table 3:** Identified chemical compounds in evolved gas derived from SADP adhesives heated at 170 °C for 60 s.

Samples	Peak Number	RT (min)	SI	Compound	CAS	MW	Formula
SADP (100/0)	1	7.47	89	4H-Pyran-4-one,2,3-dihydro-3,5-dihydroxy-6-methyl-	28564-83-2	144	C_6_H_8_O_4_
	2	8.27	95	5-Hydrxoymethylfurfura	67-47-0	126	C_6_H_6_O_3_
SADP (90/10)	1′	4.67	98	Furfural	1998-1-1	96	C_5_H_4_O_2_
			96	3-Furaldehyde	498-60-2	96	C_5_H_4_O_2_
	2′	5.66	95	2(5H)-Furanone, 5-methyl-	591-11-7	98	C_5_H_6_O_2_
	3′	5.81	98	2-Furancarboxaldehyde, 5-methyl-	620-02-0	110	C_6_H_6_O_2_
	4′	6.78	96	2,5-Furandicarboxaldehyde	823-82-5	124	C_6_H_4_O_3_
	5′	6.88	95	Furyl hydroxymethyl ketone	17678-19-2	126	C_6_H_6_O_3_
			94	Furan-2-carbohydrazide	3326-71-4	126	C_5_H_6_N_2_O_2_
			94	Methyl 2-furoate	611-13-2	126	C_6_H_6_O_3_
			94	3-Furancarboxylic acid, methyl ester	13129-23-2	126	C_6_H_6_O_3_
	6′	7.10	94	Levoglucosenone	37112-31-5	126	C_6_H_6_O_3_
	7′	7.43	94	4H-Pyran-4-one,2,3-dihydro-3,5-dihydroxy-6-methyl-	28564-83-2	144	C_6_H_8_O_4_
	8′	8.08	90	5-Acetoxymethyl-2-furaldehyde	10551-58-3	168	C_8_H_8_O_4_
	9′	8.29	96	5-Hydrxoymethylfurfura	67-47-0	126	C_6_H_6_O_3_
